# Implicit Transpositions in DCJ Scenarios

**DOI:** 10.3389/fgene.2017.00212

**Published:** 2017-12-12

**Authors:** Pavel Avdeyev, Shuai Jiang, Max A. Alekseyev

**Affiliations:** ^1^Department of Mathematics and the Computational Biology Institute, George Washington University, Washington, DC, United States; ^2^Department of Computer Science and Engineering, University of South Carolina, Columbia, SC, United States

**Keywords:** genome rearrangements, transpositions, DCJ, breakpoint graphs, chromosome evolution

## Abstract

Genome rearrangements are large-scale evolutionary events that shuffle genomic architectures. The minimal number of such events between two genomes is often used in phylogenomic studies to measure the evolutionary distance between the genomes. Double-Cut-and-Join (DCJ) operations represent a convenient model of most common genome rearrangements (reversals, translocations, fissions, and fusions), while other genome rearrangements, such as transpositions, can be modeled by pairs of DCJs. Since the DCJ model does not directly account for transpositions, their impact on DCJ scenarios is unclear. In the present work, we study implicit appearance of transpositions (as pairs of DCJs) in DCJ scenarios. We consider shortest DCJ scenarios satisfying the maximum parsimony assumption, as well as more general DCJ scenarios based on some realistic but less restrictive assumptions. In both cases, we derive a uniform lower bound for the rate of implicit transpositions, which depends only on the genomes but not a particular DCJ scenario between them. Our results imply that implicit appearance of transpositions in DCJ scenarios may be unavoidable or even abundant for some pairs of genomes. We estimate that for mammalian genomes implicit transpositions constitute at least 6% of genome rearrangements.

## 1. Introduction

Genome rearrangements are dramatic evolutionary events that change genome structures. The number of genome rearrangements between two genomes represents a good measure for their evolutionary closeness and is used as such in phylogenomic studies. This measure is often based on the maximum parsimony assumption, implying that the evolutionary distance can be estimated as the *minimum* number of rearrangements (known as the *rearrangement distance*) to transform one genome into the other. However, the maximum parsimony assumption may not always hold, inspiring the notion of the *true evolutionary distance* (Lin and Moret, 2008; Alexeev and Alekseyev, 2017).

The most common rearrangements are *reversals* that inverse contiguous segments of chromosomes, *translocations* that exchange tails of two chromosomes, and *fissions*/*fusions* that split/merge chromosomes. All these rearrangements can be conveniently modeled by Double-Cut-and-Join (DCJ) operations (Yancopoulos et al., 2005), also known as 2-breaks (Alekseyev and Pevzner, 2008), which make up to 2 “cuts” in a genome and “glue” the resulting genomic fragments in a new order.

*Transpositions* represent yet another type of genome rearrangements that relocate genomic segments across the genome. In contrast to reversal-like rearrangements modeled by DCJs (2-breaks), transpositions correspond to *3-breaks* (Alekseyev and Pevzner, 2008), which make 3 cuts and 3 gluings in a genome^1^. Transpositions are more “powerful” than reversal-like rearrangements, so in the model that includes both types of rearrangements (as 3-breaks and DCJs), the former tend to appear in shortest rearrangement scenarios in a large proportion. However, in reality transpositions happen more rarely than reversals and typically appear in a small proportion in the course of evolution (e.g., Ranz et al. 2003 estimate that in Drosophila evolution transpositions constitute less than 10% of genome rearrangements). Jiang and Alekseyev (2011) show that even the most promising model of *weighted genomic distance* (Eriksen, 2001; Bader and Ohlebusch, 2007; Fertin et al., 2009), where transpositions are assigned a higher weight, cannot bound their proportion in the corresponding optimal rearrangement scenarios to a biologically reasonable value. This emphasizes the need for an adequate model for analysis of transpositions among other types of genome rearrangements.

While a transposition cannot be directly modeled by a DCJ, it can be modeled by a pair of DCJs. We refer to such pair of DCJs as an *implicit transposition*. We remark that DCJs forming an implicit transposition may not necessarily appear consecutively in a DCJ scenario. Furthermore, two implicit transpositions may share a DCJ and thus correspond to at most one actual transposition. So we pose a question of how many transpositions can be *simultaneously* recovered from a given DCJ scenario by shuffling DCJs and replacing suitable pairs of consecutive DCJs with transpositions. We consider both *shortest* DCJ scenarios resulting from the maximum parsimony assumption, and more general *proper* DCJ scenarios based on some realistic but less restrictive assumptions. In both cases, we derive an universal lower bound for the rate of implicit transpositions, which depends only on the genomes but not a particular DCJ scenario between them. Our results imply that implicit appearance of transpositions in DCJ scenarios may be unavoidable or even abundant for some pairs of genomes.

The paper is organized as follows. In section 2, we describe graph-theoretical representation of genomes and DCJ rearrangements. In section 3, we analyze shuffling of DCJ scenarios and introduce the dependency graphs capturing their combinatorial structure. In section 4, we study the appearance of (disjoint) implicit transpositions in proper and shortest DCJ scenarios between two genomes, and prove uniform lower bounds for their rate. In section 5, we use our results to estimate the rate of implicit transpositions in DCJ scenarios between mammalian genomes and between yeast genomes. We conclude the paper with discussion in section 6.

## 2. Breakpoint graphs and DCJ scenarios

**Figure 1:**
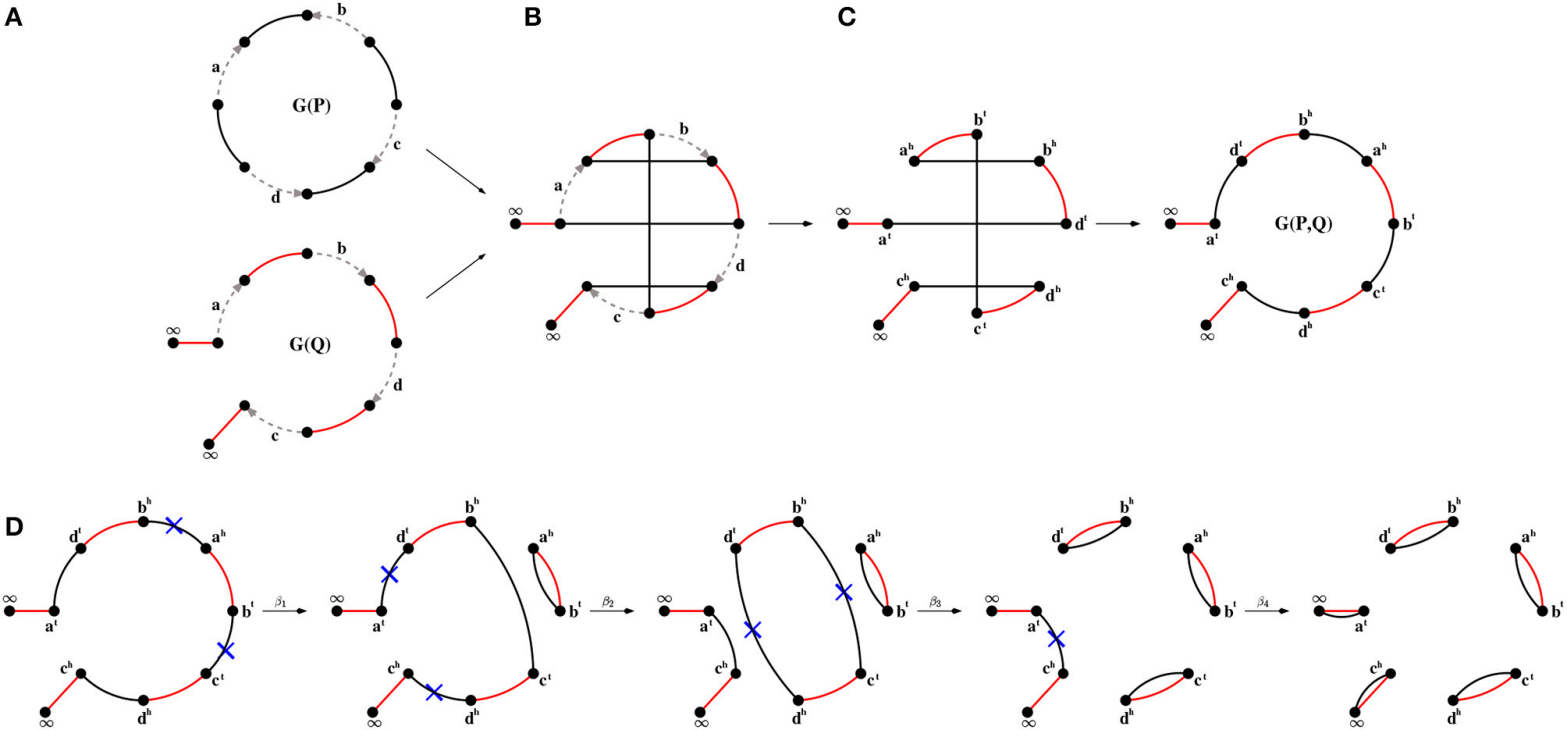
**(A)** Genome graphs *G*(*P*) and *G*(*Q*) for unichromosomal circular genome *P* = {+*a* − *b* + *c* − *d*} and unichromosomal linear genome *Q* = (+*a* + *b* + *d* + *c*), where undirected *P*-edges and *Q*-edges are colored black and red, respectively. **(B)** The superposition of genome graphs *G*(*P*) and *G*(*Q*). **(C)** The breakpoint graph *G*(*P, Q*) (two layouts) is obtained from the superposition of *G*(*P*) and *G*(*Q*) with removal of directed edges. The graph *G*(*P, Q*) is formed by a single black-red path, i.e., *p*_*even*_(*P, Q*) = 0, *c*(*P, Q*) = 0, *p*_*odd*_(*P, Q*) = 1. **(D)** A transformation of the breakpoint graph *G*(*P, Q*) into *G*(*Q, Q*), representing to a shortest DCJ scenario (of length *d*_DCJ_(*P, Q*) = 4) between genomes *P* and *Q*.

Let *P* be a genome with circular and/or linear chromosomes. We represent a circular chromosome consisting of *n* genes as a cycle with *n* directed edges (encoding genes and their strands) alternating with *n* undirected edges connecting extremities of adjacent genes. Similarly, we represent a linear chromosome consisting of *n* genes as a path with *n* directed edges alternating with *n*+1 undirected edges, where *n*−1 undirected edges connect extremities of adjacent genes and two more undirected edges connect each endpoint extremity to its own special vertex labeled ∞ (corresponding to telomeres). The genome graph *G*(*P*) is a collection of such cycles and paths (Figure 1A).

A DCJ in genome *P* mimics some genome rearrangement and corresponds to a replacement of one or two undirected edges in the genome graph *G*(*P*) in one of the following ways:

1. {*x, y*}, {*u, v*} → {*x, u*}, {*y, v*} (internal reversals, translocations),
2. {*x, y*}, {*u*, ∞} → {*x, u*}, {*y*, ∞} (reversals at chromosome ends, translocations involving a whole chromosome),
3. {*x*, ∞}, {*y*, ∞} → {*x, y*} (fusions),
4. {*x, y*} → {*x*, ∞}, {*y*, ∞} (fissions),

where *x*, *y*, *u*, *v* are regular (non-∞) vertices.

For genomes *P* and *Q* composed of the same set of genes, the *breakpoint graph G*(*P, Q*) is defined as the superposition of individual genome graphs *G*(*P*) and *G*(*Q*), and can be constructed by “gluing” the identically labeled directed edges in the graphs (Figures 1B,C). From now on, we will ignore directed edges and assume that *G*(*P, Q*) consists only of undirected edges, where the edges from genome *P* (*P-edges*) are colored black and the edges from genome *Q* (*Q-edges*) are colored red. Then the breakpoint graph *G*(*P, Q*) represents a collection of cycles and paths consisting of undirected edges alternating between black and red colors. We distinguish the following types of cycles and paths with respect to their *length* ℓ (i.e., the number of edges in a cycle or path): trivial cycles and paths (ℓ = 2), even paths (ℓ is even) and odd paths (ℓ is odd). We denote the number of cycles, trivial cycles, paths, trivial paths, even paths, and odd paths in *G*(*P, Q*) as *c*(*P, Q*), *c*_2_(*P, Q*), *p*(*P, Q*), *p*_2_(*P, Q*), *p*_even_(*P, Q*), and *p*_odd_(*P, Q*), respectively. By definition, we have *p*_even_(*P, Q*) + *p*_odd_(*P, Q*) = *p*(*P, Q*).

While at the ends of an even path there is always a *P*-edge and a *Q*-edge, an odd path starts and ends with the same edge color. We therefore will distinguish odd paths with *P*-edges at the ends (*PP-paths*) and with *Q*-edges at the ends (*QQ-paths*), and denote their number by $p_{\text{odd}}^{P}\left(P,Q\right)$ and $p_{\text{odd}}^{Q}\left(P,Q\right)$, respectively. Trivially, we have $p_{\text{odd}}\left(P,Q\right)=p_{\text{odd}}^{P}\left(P,Q\right)+p_{\text{odd}}^{Q}\left(P,Q\right)$. Since in *G*(*P, Q*) there are $p_{\text{odd}}^{P}\left(P,Q\right)+\frac{p_{\text{even}}\left(P,Q\right)}{2}$ *P*-edges incident to ∞, which corresponds to telomeres in genome *P*, the number of linear chromosomes in *P* equals $p_{\text{odd}}^{P}\left(P,Q\right)\;+\;\frac{p_{\text{even}}\left(P,Q\right)}{2}$. Similarly, in genome *Q* the number of linear chromosomes equals $p_{\text{odd}}^{Q}\left(P,Q\right)\;+\;\frac{p_{\text{even}}\left(P,Q\right)}{2}$.

A DCJ scenario transforming genome *P* into genome *Q* corresponds to a transformation of the breakpoint graph *G*(*P, Q*) into the breakpoint graph *G*(*Q, Q*),

which consists of trivial cycles and trivial paths (Figure 1D).

Reconstruction of DCJs happened in the evolution between genomes of extant species represents a challenging task in comparative genomics. Such reconstruction is often based on the parsimony assumption that evolutionary DCJs (i.e., genome rearrangements) between two genomes form a *shortest* DCJ scenario. However, in reality the parsimony assumption may not always hold, emphasizing the need to consider DCJ scenarios that are not necessarily shortest (Lin and Moret, 2008; Alexeev and Alekseyev, 2017). We consider a class of DCJ scenarios under realistic but less restrictive assumptions, which includes the class of shortest DCJ scenarios as a subclass. Namely, we call a DCJ scenario between genomes *P* and *Q proper* if in the corresponding transformation of the breakpoint graphs from *G*(*P, Q*) to *G*(*Q, Q*), the following three conditions hold:

(P1) any edge once removed is never recreated (that is, in the course of evolution, each gene adjacency is either preserved, or broken and never restored);
(P2) no pair of DCJs (not necessarily adjacent) can be replaced by an equivalent single DCJ (that is, there is no obvious way to shorten the scenario);
(P3) the number of fusions and fissions does not exceed $p_{\text{odd}}^{P}\left(P,Q\right)$ and $p_{\text{odd}}^{Q}\left(P,Q\right)$, respectively (in particular, we avoid unrealistic scenarios where one genome is cut into genes by fissions and then glued into the other genomes by fusions). Lemma 3 below states that these bounds are the maximum numbers of such rearrangements that may appear in shortest DCJ scenarios.

Below we prove that shortest DCJ scenarios satisfy these properties and thus are proper. We start with recalling and proving some useful lemmas.

THEOREM 1 (Tannier et al. 2009). *The DCJ distance between genomes P and Q on n genes equals*

$$d_{\text{DCJ}}\left(P,Q\right)=n-c\left(P,Q\right)-\frac{p_{even}\left(P,Q\right)}{2}.$$

**LEMMA 2**. (Bergeron et al., 2006)^2^. *In a shortest DCJ scenario transforming genome P into genome Q, each DCJ performs one of the following operations on P-edges in the breakpoint graph*:

- *splits a non-trivial cycle into two cycles*,
- *splits a non-trivial even path into a cycle and an even path*,
- *splits an odd path into a cycle and an odd path*,
- *closes a *PP*-path into a cycle*,
- *splits a *QQ*-path into two even paths*,
- *transforms a *PP*-path and a *QQ*-path into two even paths*.

**LEMMA 3**. *Let t be any shortest DCJ scenario transforming genome P into genome Q. Then the number of fusions and fissions in t is bounded by $p_{\text{odd}}^{P}\left(P,Q\right)$ and $p_{\text{odd}}^{Q}\left(P,Q\right)$, respectively. Furthermore, there exists a shortest DCJ scenario transforming genome P into genome Q such that it contains exactly $p_{\text{odd}}^{P}\left(P,Q\right)$ fusions and $p_{\text{odd}}^{Q}\left(P,Q\right)$ fissions*.

*Proof*. Let *t* be any shortest DCJ scenario transforming a genome *P* into a genome *Q*. Fusions and fissions in *t* correspond to DCJs of type (iv) and (v) as defined in Lemma 2. Hence, every fusion eliminates one *PP*-path and every fission eliminate one *QQ*-path in the breakpoint graph. Lemma 2 also implies that the number of *PP*-paths and the number of *QQ*-paths never increases along *t*. Hence, the number of fusions and fissions in *t* is bounded by $p_{\text{odd}}^{P}\left(P,Q\right)$ and $p_{\text{odd}}^{Q}\left(P,Q\right)$, respectively.

It it easy to construct a shortest DCJ scenario that uses DCJs of types (i), (ii), (iv), (v) only. Indeed, these types of DCJs define how to process existing connected components in the breakpoint graph until they all turn into trivial path/cycles. Such scenario eliminates *PP*-paths and *QQ*-paths with fusions and fissions. So, it must contain exactly $p_{\text{odd}}^{P}\left(P,Q\right)$ fusions and $p_{\text{odd}}^{Q}\left(P,Q\right)$ fissions.           □

Now, we are ready to prove that any shortest DCJ scenario is proper.

**THEOREM 4**. *Any shortest DCJ scenario between two genomes is proper*.

*Proof*. Let *t* be any shortest DCJ scenario between two genomes. Lemma 3 implies that *t* satisfies the condition (P3) of a proper DCJ scenario. It is also clear that *t* satisfies the condition (P2) as otherwise we would be able to shorten it.

To prove the condition (P1), we notice that if an edge (*u, v*) is removed from the breakpoint graph by a DCJ in *t*, then by Lemma 2 after this DCJ vertices *u* and *v* start to belong to distinct paths/cycles and at least one of the vertices *u* and *v* belongs to a cycle or an even path. Again by Lemma 2, no subsequent DCJ in *t* can make these vertices to belong to the same cycle or path again. That is, the edge (*u, v*) is never re-created.                □

## 3. Shuffling of DCJ scenarios and dependency graphs

Recall that each DCJ removes and adds some edges in a breakpoint graph. Two adjacent DCJs α and β in a DCJ scenario are called *independent* if β removes edges that were not created by α. Otherwise, if β removes some edge(s) created by α, then β *depend*s on α. Furthermore, let *k* ∈ {1, 2} be the number of edges created by α and removed by β. We say that β *strongly depends* on α if *k* = 2, and *weakly depends*^3^ on α if *k* = 1. We remark that proper DCJ scenarios cannot contain strongly dependent DCJs by the condition (P2).

In a DCJ scenario, one can change the order of two adjacent independent DCJs and obtain another DCJ scenario of the same length between the same two genomes. Similarly, a pair of adjacent weakly dependent DCJs in a DCJ scenario can be replaced with another pair of weakly dependent DCJs, resulting in a new DCJ scenario of the same length between the same two genomes (Braga and Stoye, 2010; Jiang and Alekseyev, 2014).

We therefore consider the following two types of length-preserving operations, which can be applied to a pair of adjacent DCJs (α, β) in a DCJ scenario:

(T1) If α and β are independent, replace (α, β) with (β, α).
(T2) If α and β are weakly dependent, replace (α, β) with an equivalent pair of weakly dependent DCJs.

**Figure 2:**
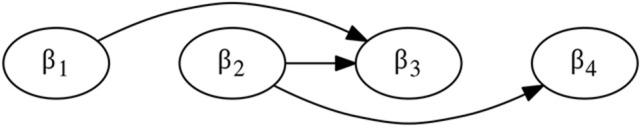
The dependency graph DG(*t*) for DCJ scenario *t* defined in Figure 1D.

To better capture and analyze the combinatorial structure of DCJs in a DCJ scenario *t*, we construct the *dependency graph* DG(*t*) (also called *overlap graph* in Ozery-Flato and Shamir 2006; Ouangraoua and Bergeron 2010), whose vertices are labeled with DCJs from *t* and there is an arc (α, β) whenever β depends on α (Figure 2).

**THEOREM 5**. *Let t be a proper DCJ scenario between two genomes composed of the same genes. Then*

- *the pairs of dependent DCJs in t are in one-to-one correspondence with the arcs in DG(t)*;
- *both indegree and outdegree of each vertex in DG(t) are at most 2*;
- *t represents a topological ordering of DG(t)*;
- *DG(t) is acyclic*.

*Proof*. An arc (α, β) in DG(*t*) corresponds in the breakpoint graph transformation *t* to the edge that is created by DCJ α and removed by DCJ β. Since *t* is proper, the removed edges are never recreated, implying that this correspondence is one-to-one.

Furthermore, any DCJ in *t* (which removes at most two edges and creates at most two edges) depends on at most two other DCJs and may have at most two weakly dependent DCJs. That is, both indegree and outdegree of any vertex in DG(*t*) are bounded by 2.

If (α, β) is an arc in DG(*t*), then DCJ β removes some edge *e* created by DCJ α. No other DCJ besides α can create *e* because *t* is a proper transformation. Thus β must follow α in *t*. So *t* represents a topological ordering for DG(*t*) and therefore DG(*t*) is acyclic.                □

Braga and Stoye (2010) show that any shortest DCJ scenario can be obtained from any other shortest DCJ scenario between the same two genomes using only operations of types (T1) and (T2). The following theorem extends this result to proper DCJ scenarios and operations (T1) only.

**THEOREM 6**. *Let t_1_ and t_2_ be proper DCJ scenarios between the same two genomes. Scenario t_1_ can be obtained from scenario t_2_ with operations (T1) if and only if* DG(*t*_1_) = DG(*t*_2_).

*Proof*. Suppose that *t*_1_ and *t*_2_ correspond to the same dependency graph, i.e., DG(*t*_1_) = DG(*t*_2_) = *G*. Then by Theorem 5 *t*_1_ and *t*_2_ represent topological orderings of *G*. We will show that *t*_1_ and *t*_2_ can be obtained from each other with operations (T1). Suppose that *t*_1_ and *t*_2_ start with the same *k* DCJs but then diverge, i.e., *t*_1_ = (α_1_, α_2_, …, α_*k*_, γ, …) and *t*_2_ = (α_1_, α_2_, …, α_*k*_, β_1_, β_2_, …, β_*m*_, γ, …), where γ≠β_1_ are the first DCJs different in the two scenarios. We will show that γ in *t*_2_ can be moved to (*k* + 1)-st position (i.e., its position in *t*_1_) with operations (T1). Since β_*m*_ follows γ in *t*_1_ but precedes γ in *t*_2_, these vertices are not connected with an arc in *G* and we can apply operation (T1) to *t*_2_ to obtain (α_1_, α_2_, …, α_*k*_, β_1_, β_2_, …, γ, β_*m*_, …). After *m* such operations we get (α_1_, α_2_, …, α_*k*_, γ, β_1_, β_2_, …, β_*m*_, …), where γ is at the same position as in *t*_1_. Using induction on *k*, we conclude that *t*_1_ can be obtained from *t*_2_ with operations (T1), and vice versa.

Now, suppose that DCJ scenarios *t*_1_ and *t*_2_ can be obtained from each other with operations (T1). Since operations (T1) changes only the order of DCJs in the scenario but keeps the DCJs themselves intact, the dependency graph is not affected by such operations either. Therefore, DG(*t*_1_) = DG(*t*_2_).                □

Let *e*(*t*) be the number of arcs in DG(*t*). We will need the following lower bound of *e*(*t*) for any proper DCJ scenario *t*.

**THEOREM 7**. *Let t be a proper DCJ scenario between genomes P and Q composed of the same n genes. Then the number of arcs in DG(t) is bounded as follows*:

$$e\left(t\right)\geq 2|t|-n-p_{odd}\left(P,Q\right)-\frac{p_{even}\left(P,Q\right)}{2}+c_{2}\left(P,Q\right)+p_{2}\left(P,Q\right).$$

*Proof*. It is easy to see that the number of *P*-edges in the breakpoint graph *G*(*P, Q*) equals $n+p_{\text{odd}}^{P}\left(P,Q\right)+\frac{p_{\text{even}}\left(P,Q\right)}{2}$. Among them exactly $m\left(P,Q\right)=n+p_{\text{odd}}^{P}\left(P,Q\right)+\frac{p_{\text{even}}\left(P,Q\right)}{2}-c_{2}\left(P,Q\right)-p_{2}\left(P,Q\right)$ *P*-edges belong to the non-trivial cycles/paths in *G*(*P, Q*). These *P*-edges have to be removed by DCJs in *t* in order to form trivial cycles or paths in *G*(*Q, Q*). The other *P*-edges removed by DCJs in *t* must have been created by earlier DCJs. By the definition of a proper scenario, at most $p_{\text{odd}}^{Q}\left(P,Q\right)$ DCJs in *t* remove one *P*-edge, while the other DCJs remove two *P*-edges. Thus, the total number of removed *P*-edges by DCJs in *t* is at least $2|t|\;-\;p_{\text{odd}}^{Q}\left(P,Q\right)$. The number of previously created and then removed *P*-edges is therefore at least

$$\begin{array}{l} 2|t|-p_{\text{odd}}^{Q}\left(P,Q\right)-m\left(P,Q\right)=2|t|-n\\ -p_{\text{odd}}\left(P,Q\right)-\frac{p_{\text{even}}\left(P,Q\right)}{2}+c_{2}\left(P,Q\right)+p_{2}\left(P,Q\right) \end{array}$$

which gives a lower bound for the number of arcs in DG(*t*) by Theorem 5.                □

From Theorem 1 and 7, we easily get the following statement:

COROLLARY 8. *Let t be a shortest DCJ scenario between genomes P and Q composed of the same n genes. Then e(t) ≥ E(P, Q), where*

$$\begin{array}{l} E\left(P,Q\right)=n-2\cdot c\left(P,Q\right)-p\left(P,Q\right)-\frac{p_{\text{even}}\left(P,Q\right)}{2}\\ +\;c_{2}\left(P,Q\right)+p_{2}\left(P,Q\right). \end{array}$$

## 4. Implicit transpositions in DCJ scenarios

While DCJs mimic most common genome rearrangements (reversals, translocations, fissions, fusions), more complex rearrangements such as transpositions cannot be modeled by a single DCJ. A transposition, which cuts off a segment of a chromosome and inserts it into some other place in the genome, can be modeled by a pair of weakly dependent DCJs, replacing three undirected edges with three other undirected edges on the same six vertices in the genome graph. We remark that this graph operation is also known as a 3-break rearrangement (Alekseyev and Pevzner, 2008).

Below we study how transpositions appearing in the course of evolution between two genomes may be captured by DCJ scenarios between these genomes. While a transposition constitutes a pair of DCJs, their positions in a DCJ scenario may not always be reconstructed correctly. In particular, the two DCJs forming a transposition may appear interweaved with other independent DCJs that precede or follow this transposition in the course of evolution. This inspires the following definition.

In a DCJ scenario *t* = (α_1_, α_2_, …, α_*n*_), a pair of weakly dependent DCJs (α_*i*_, α_*j*_) forms an *implicit transposition* if these DCJs can be made adjacent by applying a number of operations (T1). Such adjacent DCJs then can be replaced by a single transposition (modeled by a 3-break). We refer to such a transposition as *recovered* from the DCJ scenario *t*. This poses us a question of how many transpositions can be *simultaneously* recovered from a given proper DCJ scenario *t*.

Since two distinct implicit transpositions in a proper DCJ scenario *t* may share a DCJ, the maximum number of transpositions that can be recovered from *t* may be smaller than the number of implicit transpositions in *t*. We therefore are interested in (pairwise) *disjoint* implicit transpositions, which do not share any DCJs between them. Furthermore, it is not immediately clear if existence of a set of *m* disjoint implicit transpositions in *t* implies that all these *m* transpositions can be simultaneously recovered from *t*. We will prove below that this is indeed the case. We therefore define DIT(*t*) as the maximum number of disjoint implicit transpositions in *t*, which, as we will show, also equals the maximum number of transpositions that can be simultaneously recovered from *t*.

Simultaneously recovering DIT(*t*) transpositions from *t*, we will obtain a scenario of length |*t*| − DIT(*t*) composed of DIT(*t*) transpositions and |*t*| − 2 · DIT(*t*) DCJs. The proportion of transpositions in this scenario is $r\left(t\right)=\frac{\text{DIT}\left(t\right)}{|t|-\text{DIT}\left(t\right)}$, which we refer to as the *rate of implicit transpositions* in *t*. Since there exist many different proper DCJ scenarios between two genomes, our goal will be to derive a lower bound for *r*(*t*) that does not depend on *t*, but only on the given genomes.

### 4.1. Disjoint implicit transpositions as matchings

From the definition of an implicit transposition it follows that an implicit transposition formed by a pair of DCJs (α, β) in a proper DCJ scenario *t* corresponds to an arc in the dependency graph DG(*t*). However, it is not immediately clear if every arc (*x, y*) in DG(*t*) represents an implicit transposition, i.e., if DCJs *x* and *y* in *t* can be made adjacent with operations (T1). We call an arc (*x, y*) a *shortcut* if there exists a path between vertices *x* and *y* in DG(*t*) that does not pass through (*x, y*). We will show that shortcuts represents the only case for making DCJs adjacent with operations (T1).

**THEOREM 9**. *Let G be a directed acyclic graph. An arc (α_1_, α_2_) is a shortcut in G if and only if there does not exist no topological ordering of G in which α_1_ and α_2_ are adjacent*.

*Proof*. From the definition of a shortcut, it follows that its endpoints cannot be adjacent in any topological ordering of *G*.

Now, we prove that if an arc (α_1_, α_2_) is not a shortcut then there exists a topological ordering of *G*, such that endpoints of arc (α_1_, α_2_) are adjacent. Let *t* = (β_1_, …, β_*k*_, α_1_, γ_1_, …, γ_*m*_, α_2_, δ_1_, …, δ_*w*_) be any topological ordering of *G*. Let *S* be the set containing vertices γ_*i*_ such that there is a directed path from α_1_ to γ_*i*_, and let *T* = {γ_1_, γ_2_, …, γ_*m*_} \ *S*. It follows that there is no path from a vertex α_1_ to a vertex *x* ∈ *T* and no path from *y* ∈ *S* to α_2_. Hence, we construct a new topological ordering $t^{\prime }=\left(\beta_{1},\ldots ,\beta_{k},T,\alpha_{1},\alpha_{2},S,\delta_{1},\ldots ,\delta_{w}\right)$, where vertices from *T* and *S* in *t*′ appear in the same order as in *t*. We constructed a topological ordering of *G*, where endpoints of arc (α_1_, α_2_) are adjacent.                □

**THEOREM 10**. *Let G be a directed acyclic graph. Then for any matching M in G that does not contain shortcuts, there exists a topological ordering t of G such that for any arc (α_1_, α_2_) ∈ M, DCJs α_1_ and α_2_ are adjacent in t*.

*Proof*. We prove the theorem statement by induction on |*M*|. For the base case |*M*| = 1, the statement follows from Theorem 9. Assume now that the statement holds for |*M*| = *m* ≥ 1.

Let |*M*| = *m* + 1, (α_1_, α_2_) be an arc in *M*, and *M*′ = *M* \ {(α_1_,α_2_)}. Let *G*′ be a graph obtained from *G* by removing arc (α_1_, α_2_) and gluing vertices α_1_, α_2_ into a new single vertex β. Since the arc (α_1_, α_2_) is not a shortcut, such contraction of arc (α_1_, α_2_) cannot created a cycle in *G*′. Hence, *G*′ is a directed acyclic graph and matching *M*′ in *G*′ does not contain shortcuts. Since |*M*′| = *m*, by the induction assumption there is a topological ordering *t*′ of *G*′ such that for any arc $\left(\gamma_{1},\gamma_{2}\right)\in M^{\prime }$, γ_1_ and γ_2_ are adjacent in *t*′.

We obtain *t* from *t*′ by replacing the vertex β with the ordered pair of vertices α_1_, α_2_. It is easy to see that such *t* represents the required topological ordering for *G*.                □

For a directed graph *G*, we define $\bar{G}$ as the undirected graph obtained from *G* by making all arcs undirected. We call a graph *G* a *directed forest* if $\bar{G}$ is a forest. We will need the following lemma about a lower bound of matching size in a directed forest:

**LEMMA 11**. *Let G be a directed forest such that the degree of each vertex is bounded by d. Then there exists a matching M in G such that*

$$|M|\geq \left\lceil \frac{e\left(G\right)}{d}\right\rceil ,$$

*where e(G) is the number of arcs in G*.

*Proof*. Let us construct a matching *M* in $\bar{G}$ iteratively. Initially we let *M* = ∅. If $\bar{G}$ contains at least one edge, it also contains a leaf α (i.e., vertex of degree 1). We add its only incident edge (α, β) to *M* and remove from $\bar{G}$ all edges incident to the vertex β. Since degree of each vertex in $\bar{G}$ is bounded by *d*, at most *d* such edges are deleted. We iterate this procedure until all edges of $\bar{G}$ are removed. Thus we perform at least $\left\lceil \frac{e\left(\bar{G}\right)}{d}\right\rceil$ iterations, implying that $|M|\geq \left\lceil \frac{e\left(\bar{G}\right)}{d}\right\rceil$. By construction, it is clear that *M* forms a matching in $\bar{G}$ and thus under a suitable orientation of the edges in *M*, it also forms a matching in *G*.                □

### 4.2. Implicit transpositions in proper DCJ scenarios

We will need the following lemma.

**LEMMA 12**. *Let t be a proper DCJ scenario between two genomes composed of the same genes, and M be a matching in DG(t) with no shortcuts. Then*

DIT(t)≥|M|.

*Proof*. By Theorem 10, there exists a topological ordering *t*′ of DG(*t*) such that the endpoints of all arcs from *M* are adjacent in *t*′. By Theorem 6, the topological ordering *t*′ can be obtained from *t* with operations (T1), implying that one can simultaneously recover from *t* all arc (representing pairs of weakly dependent DCJs in *t*) present in *M*. Therefore, DIT(*t*) ≥ |*M*|.                □

**THEOREM 13**. *Let t be a proper DCJ scenario between two genomes composed of the same genes. Then*

$$\text{DIT}\left(t\right)\geq \lceil \frac{e\left(t\right)}{6}\rceil .$$

*Proof*. By Theorem 5, the graph DG(*t*) is acyclic. Let *V* be the vertex set of DG(*T*) and *V*_*l*_ be the set of vertices *v* ∈ *V* such that the longest path from a source (i.e., a vertex of indegree 0) to *v* has length *l*. In particular, *V*_0_ contains all the sources. Let *k* = |*V*_0_| be the number of sources.

From the definition, it follows that each vertex from *V*_*l*_ for *l* > 0 has at least one incoming arc starting at a vertex from *V*_*l*−1_. Let us fix one such incoming arc for each vertex from *V*_*l*_, and consider the subgraph *G* obtained from DG(*t*) by removing all arcs except the fixed ones. Then *G* contains |*V*| vertices and |*V*| − *k* arcs. Since by Theorem 5 the indegree and outdegree of each vertex in DG(*t*) are at most 2, the degree of each vertex in *G* is at most 3. Then by Lemma 11, there exists a matching *M* in *G* without shortcuts such that $|M|\geq \left\lceil \frac{|V|-k}{3}\right\rceil$. Furthermore, since DG(*t*) contains at most 2(|*V*| − *k*) arcs, we have $|M|\geq \frac{2\left(|V|-k\right)}{6}\rceil \geq \lceil \frac{e\left(t\right)}{6}\rceil$.

From the definition of *G*, it follows that each arc from *G* does not form a shortcut in DG(*t*). Indeed, for each arc (*u, v*) from *G*, we have *u* ∈ *V*_*l*−1_ and *v* ∈ *V*_*l*_ for some *l* > 0. If (*u, v*) forms a shortcut in DG(*t*), then there exists a path between *u* and *v* of length greater than 1, implying that there exists a path from a source to *v* of length greater than *l*, which contradicts the condition *v* ∈ *V*_*l*_. Therefore, *M* represents a matching in DG(*t*) without shortcuts, and thus by Lemma 12, $\text{DIT}\left(t\right)\geq |M|\geq \lceil \frac{e\left(t\right)}{6}\rceil$.                □

The following theorem gives a uniform lower bound for *r*(*t*), which does not depend on a particular scenario *t*.

COROLLARY 14. *Let t be any proper DCJ scenario between genomes P and Q composed of the same n genes. Then*

$$r\left(t\right)\geq \frac{1}{2}-\frac{3s\left(P,Q\right)}{8d_{\text{DCJ}}\left(P,Q\right)+2s\left(P,Q\right)},$$

*where $s\left(P,Q\right)=n+p_{odd}\left(P,Q\right)+\frac{p_{even}\left(P,Q\right)}{2}-c_{2}\left(P,Q\right)-p_{2}\left(P,Q\right)$*.

*Proof*. Since *n* ≥ *c*_2_(*P, Q*) + *p*_2_(*P, Q*), we have *s*(*P, Q*) ≥ 0. By Theorems 7 and 13, we have

$$\begin{array}{cc} r\left(t\right) & =\frac{\text{DIT}\left(t\right)}{|t|-\text{DIT}\left(t\right)}\geq \frac{\frac{e\left(t\right)}{6}}{|t|-\frac{e\left(t\right)}{6}}\geq \frac{2|t|-s\left(P,Q\right)}{4|t|+s\left(P,Q\right)}\\ =\frac{1}{2}-\frac{3s\left(P,Q\right)}{8|t|+2s\left(P,Q\right)}\geq \frac{1}{2}-\frac{3s\left(P,Q\right)}{8d_{\text{DCJ}}\left(P,Q\right)+2s\left(P,Q\right)}. \end{array}$$

               □

### 4.3. Implicit transpositions in shortest DCJ scenarios

In this section we focus on shortest DCJ scenarios, which represent a special case of proper DCJ scenarios. For shortest DCJ scenarios, we can refine the uniform lower bound for the rate of implicit transposition given in Corollary 14.

Let *t* be a shortest DCJ scenario between two genomes. While Theorem 5 claims that DG(*t*) is acyclic, the results of Shao et al. (2013) imply that $\bar{\text{DG}\left(t\right)}$ is a forest^4^:

THEOREM 15 (Shao et al. 2013). *Let t be a shortest DCJ scenario between two genomes composed of the same genes. Then the graph $\bar{\text{DG}\left(t\right)}$ is a forest*.

By Theorem 15, DG(*t*) is a directed forest for any shortest DCJ scenario *t*. This allows us to refine the result of Theorem 13 as follows:

**THEOREM 16**. *Let t be a shortest DCJ scenario between two genomes composed of the same genes. Then*

$$\text{DIT}\left(t\right)\geq \left\lceil \frac{e\left(t\right)}{4}\right\rceil .$$

*Proof*. By Theorem 5, the degree of each vertex in DG(*t*) is bounded by 4. By Theorem 15, $\bar{\text{DG}\left(t\right)}$ is a forest. Hence, by Lemma 11, there is a matching *M* such that $|M|\geq \lceil \frac{e\left(t\right)}{4}\rceil$. Since any arc in directed forest is not a shortcut, *M* represents a matching with no shortcuts in DG(*t*). By Lemma 12, we have $\text{DIT}\left(t\right)\geq |M|\geq \lceil \frac{e\left(t\right)}{4}\rceil$.                □

Similarly to Corollary 14, from Theorem 16 we can immediately derive a better lower bound for *r*(*t*) for any shortest DCJ scenario *t*.

COROLLARY 17. *Let t be any shortest DCJ scenario between genomes P and Q composed of the same genes. Then*

$$r\left(t\right)\geq \frac{\lceil \frac{E\left(P,Q\right)}{4}\rceil }{d_{\text{DCJ}}\left(P,Q\right)-\left\lceil \frac{E\left(P,Q\right)}{4}\right\rceil }.$$

*Proof*. Since $r\left(t\right)=\frac{\text{DIT}\left(t\right)}{d_{\text{DCJ}}\left(P,Q\right)-\text{DIT}\left(t\right)}=\frac{d_{\text{DCJ}}\left(P,Q\right)}{d_{\text{DCJ}}\left(P,Q\right)-\text{DIT}\left(t\right)}-1$, the value of *r*(*t*) monotonically increases as DIT(*t*) grows. The stated bounds for *r*(*t*) immediately follow from Theorem 16 and Corollary 8.                □

## 5. Evaluation

In this section, we estimate the rate of implicit transpositions recovered from pairwise DCJ scenarios between mammalian genomes, and between yeast genomes. For each pair of genomes, we use Corollary 14 and Corollary 17 for proper and shortest DCJ scenarios, respectively, to compute the lower bound for the rate of disjoint implicit transpositions between these genomes.

### 5.1. Mammalian genomes

We analyze a set of three mammalian genomes: *rat, macaque*, and *human* represented as sequence of 1,360 synteny blocks (Ma et al., 2006). The transposition rate between these genomes was recently estimated with statistical method (Alexeev et al., 2015). Since this method is currently limited to circular chromosomes, we artificially circularize each chromosome in the genomes and calculate the rate of implicit transpositions for these circularized genomes, in addition to original (linear) genomes.

The results in Table 1 show that the lower bound is consistent with the estimated transposition rate for circularized genomes.

**Table 1:** Lower bounds for the rate of disjoint implicit transpositions between pairs of mammalian genomes among *rat, macaque*, and *human*.

**Genome pair**	**DCJ distance**	**Lower bound for *r*(*t*), where *t* is proper**	**Lower bound for *r*(*t*), where *t* is shortest**	**Estimated rate of transpositions (Alexeev et al., 2015)**
Human and macaque	106	0.06:0.06	0.09:0.10	0.25
Human and rat	707	0.10:0.11	0.15:0.17	0.26
Macaque and rat	701	0.09:0.10	0.15:0.17	0.28

*In the entry x:y, x gives a bound for original (linear) genomes, and y gives a bound for their circularized versions*.

### 5.2. Yeast genomes

We also analyze a set of five yeast genomes: *A. gossypii, K. lactis, K. thermotolerans, S. kluyveri*, and *Z. rouxii*, represented as sequences of the same 710 synteny blocks (Chauve et al., 2010). Table 2 demonstrates that the rate of implicit transpositions in DCJ scenarios between yeast genomes is at least 0.06.

**Table 2:** Lower bounds for the rate of disjoint implicit transpositions between pairs of yeast genomes among *A. gossypii* (Ago), *K. lactis* (Kla), *K. thermotolerans* (Kth), *S. kluyveri* (Skl), *Z. rouxii* (Zro).

**Genome pair**	**DCJ distance**	**Lower bound for *r*(*t*), where *t* is proper**	**Lower bound for *r*(*t*), where *t* is shortest**
Ago and Kla	359	0.15	0.25
Ago and Kth	247	0.14	0.23
Ago and Skl	215	0.13	0.20
Ago and Zro	317	0.14	0.23
Kla and Kth	272	0.14	0.23
Kla and Skl	238	0.12	0.20
Kla and Zro	342	0.14	0.24
Kth and Skl	69	0.06	0.11
Kth and Zro	193	0.11	0.17
Skl and Zro	158	0.10	0.15

## 6. Conclusion

The present work continues the study of the combinatorial structure of DCJ scenarios from the perspective of simple shuffling operations, each affecting only a pair of consecutive DCJs (first introduced in Braga and Stoye 2010).

Recently it was shown (Jiang and Alekseyev, 2014) that any shortest DCJ scenario between a genome with *m* ≥ 1 circular chromosomes and a linear genome (consisting of linear chromosomes) can be transformed this way into a shortest DCJ scenario, where circular chromosomes are eliminated by the first *m* DCJs and the rest represents a scenario between linear genomes. This construction was further used to obtain an approximate solution for the linear genome median problem.

In the present work, we study how evolutionary transpositions may implicitly appear in DCJ scenarios and prove a uniform lower bound for their rate. Since transpositions are rather powerful rearrangements, it is not surprising that they may appear in a significant proportion that cannot be easily bounded in rearrangement scenarios between some genomes. Even though we do not yet have a recipe for limiting the effect of transpositions in the combined DCJ (2-break) and 3-break model (for which the weighting approach was proved to be a failure by Jiang and Alekseyev 2011), our present study provides a step towards better understanding of the properties of transpositions and how they may affect reconstruction of the evolutionary history.

Our analysis of mammalian genomes demonstrates that the lower bound for the (disjoint) implicit transposition rate is consistent with the estimation for the transposition rate obtained with statistical methods (Alexeev et al., 2015).

In the future work, we plan to extend our method to support other evolutionary events such as gene deletions/insertions and duplications. This will increase the accuracy and make the method applicable to genomes (such as plants) whose evolutionary history is rich in such events.

## Author contributions

All authors listed have made a substantial, direct and intellectual contribution to the work, and approved it for publication.

### Conflict of interest statement

The authors declare that the research was conducted in the absence of any commercial or financial relationships that could be construed as a potential conflict of interest.

## Footnotes

^1^While not all 3-breaks represent transpositions, they provide a convenient model for analysis of transpositions and other transposition-like rearrangements. In the present study, we adopt this model and commonly refer to 3-breaks as (generalized) transpositions.

^2^Bergeron et al. (2006) deals with *adjacency graphs*, which are dual to breakpoint graphs. Odd paths and even paths in the breakpoint graph correspond in the adjacency graph to even paths and odd paths, respectively.

^3^Such DCJs are called *enchained* in Braga and Stoye (2010).

^4^The study (Shao et al., 2013) considers more general *trajectory graphs*, from which the dependency graphs can be obtained by contraction of edges.
